# Biochemical, Transcriptional and Translational Evidences of the Phenol-*meta*-Degradation Pathway by the Hyperthermophilic *Sulfolobus solfataricus* 98/2

**DOI:** 10.1371/journal.pone.0082397

**Published:** 2013-12-11

**Authors:** Alexia Comte, Pierre Christen, Sylvain Davidson, Matthieu Pophillat, Jean Lorquin, Richard Auria, Gwenola Simon, Laurence Casalot

**Affiliations:** 1 Aix Marseille Université, CNRS/INSU, IRD, Mediterranean Institute of Oceanography (MIO), UM 110, Marseille, France; 2 Université du Sud Toulon-Var, CNRS/INSU, IRD, Mediterranean Institute of Oceanography (MIO), UM 110, La Garde, France; 3 Institut Paoli-Calmettes, Aix Marseille Université, CNRS, UMR7258, Centre de Recherche en Cancérologie de Marseille (CRCM), Marseille, France; University of Groningen, The Netherlands

## Abstract

Phenol is a widespread pollutant and a model molecule to study the biodegradation of monoaromatic compounds. After a first oxidation step leading to catechol in mesophilic and thermophilic microorganisms, two main routes have been identified depending on the cleavage of the aromatic ring: *ortho* involving a catechol 1,2 dioxygenase (C12D) and *meta* involving a catechol 2,3 dioxygenase (C23D). Our work aimed at elucidating the phenol-degradation pathway in the hyperthermophilic archaea *Sulfolobus solfataricus* 98/2. For this purpose, the strain was cultivated in a fermentor under different substrate and oxygenation conditions. Indeed, reducing dissolved-oxygen concentration allowed slowing down phenol catabolism (specific growth and phenol-consumption rates dropped 55% and 39%, respectively) and thus, evidencing intermediate accumulations in the broth. HPLC/Diode Array Detector and LC-MS analyses on culture samples at low dissolved-oxygen concentration (DOC  =  0.06 mg.L^−1^) suggested, apart for catechol, the presence of 2-hydroxymuconic acid, 4-oxalocrotonate and 4-hydroxy-2-oxovalerate, three intermediates of the *meta* route. RT-PCR analysis on oxygenase-coding genes of *S. solfataricus* 98/2 showed that the gene coding for the C23D was expressed only on phenol. In 2D-DIGE/MALDI-TOF analysis, the C23D was found and identified only on phenol. This set of results allowed us concluding that *S. solfataricus* 98/2 degrade phenol through the *meta* route.

## Introduction

Phenol is an aromatic-ring-containing compound largely used in organic chemical industry mostly for the production of bisphenol A, or as intermediate in resins, fibers, paints or pharmaceutical syntheses [1]. It also has applications in the perfumery, molecular-biology or medicine fields. Besides, it is considered as relatively dangerous for health, classified in the European Union as a mutagenic agent, with a threshold-value exposure for man set at 5 ppm in France. It is all the more harmful because of its high volatility and/or diffusivity providing a rapid propagation in the environment. Some authors have reviewed technologies used for phenol removal from wastewaters and gaseous streams [1]. A great number of microorganisms are able to degrade phenol and use it as sole energy or carbon source [2], [3]. The aerobic biodegradation has received an increasing interest in the last few decades. Phenol has been chosen as a model molecule to study the aromatic-ring fission [4]. For monoaromatic compounds, such as phenol, benzoate, salicylate, benzene, etc., the first key degradation step involves its oxidation to catechol by a monooxygenase [5], [6]. Then, catechol is degraded via two alternative pathways, depending on the microorganism [4]. In the *ortho* route, aromatic ring is cleaved between the hydroxyl groups by the catechol 1,2 dioxygenase (C12D), leading to the *cis*,*cis* muconic acid. In the *meta* route, the ring cleavage occurs next to the two hydroxyl groups (Figure S1). It is catalyzed by the catechol 2,3 dioxygenase (C23D), and leads to the 2-hydroxymuconic semialdehyde (2-HMS) [7], [8]. Then, the 2-HMS can be degraded either through the hydrolytic route or the 4-oxalocrotonate (4-OC) route [9], [10], [11]. Both *ortho* and *meta* routes can be active for the same microorganism depending on the substrate. For example, only the *ortho* route was involved on salicylate while both routes were activated on benzoate in *Pseudomonas cepacia* [12]. It can also depend on the aromatic concentration as demonstrated, in *Pseudomonas putida* grown on benzoate. At low concentrations (< 200–300 mg.L^−1^), only the *ortho* pathway is involved, while both degradation routes are activated at higher concentrations [10], [13]. In the latter case, a proteomic study showed the simultaneous presence of 8 catabolic enzymes: 3 corresponding to the *ortho*-cleavage route and 5 involved in the *meta*-cleavage one [10].

If most phenol biodegradation studies concerned mesophilic microorganisms, some involved thermophilic bacteria from the *Bacillus* genus [14], [15] or hyperthermophilic archaea [16]–[18]. In the past two decades, some reviews have dealt with the promising future of thermophile and hyperthermophile enzymes for industrial applications: thermo-stable amylases, xylanases, proteases or DNA polymerases for potential use in food, chemical or pharmaceutical industries [19]–[21]. Besides their stability at high temperature, these enzymes are also known to withstand denaturant or acidic/alkaline conditions. Moreover, they are highly specific, robust and can be produced through either fermentation by the thermophilic microorganism or by cloning in fast-growing mesophiles by DNA recombinant technology [22]. Most of the thermophilic microorganisms belong to the Archaea group, grow at low pH and usually live in extreme environments such as solfataric fields or submarine hydrothermal areas [23]. Their physiological characteristics and the general features of their genome sequences have been reviewed elsewhere [24].

Oxygen availability is an important parameter in phenol aerobic biodegradation [25]–[27] especially at high temperature (80°C), for which its solubility in water is only 3 mg.L^−1^. Viggor et al. [6] showed that oxygen was a co-substrate of the monooxygenase, responsible for the oxidation of phenol to catechol. In thermophilic *Bacilli*, it has been shown that maximal degradation was reached at an O_2_ delivery of 1 vvm, while inactivation of the C23D was observed at high O_2_ levels [28]. This limiting effect at low O_2_ levels was also observed with *Pseudomonas* CF600 together with the accumulation in the medium of 2-HMS, an intermediate of the *meta* pathway [27].

Amongst high-temperature tolerant *Archaea*, our interest has focused on *Sulfolobus solfataricus* 98/2, which genome has been sequenced [29]. Its ability to grow on phenol was recently discussed [17], [30], and kinetic parameters of phenol biodegradation were established [18]. Moreover, a C23D gene was identified in the 98/2 strain [31].

In this work, experiments were designed to study phenol-degradation route in *S. solfataricus* 98/2 cultivated in a fed-batch bioreactor. Growth, substrate and oxygen consumptions as well as product accumulated in the broth and CO_2_ production are monitored. Transcriptomic and proteomic studies are also carried out in order to determine the metabolic pathway used for phenol degradation.

## Materials and Methods

### Strain and medium

*S. solfataricus* 98/2 was used in this study [32]. Cells were maintained at -80°C and reactivated on the mineral medium reported elsewhere [17]. The strain was previously adapted to phenol by repeated batches at concentrations up to 400 mg.L^−1^, shown to be well tolerated by the strain [17], [18].

### Experimental set up

*S. solfataricus* was batch-cultivated in a 2.7 L reactor described in a previous paper [17]. Working volume was 1.8 L. The fermentor was equipped with pH, redox, dissolved-oxygen and temperature probes connected to an automat (Wago, France). The automat was connected to a computer for process monitoring and data capture (BatchPro Software, Decobecq Automatismes, France).

### Experimental conditions

*S. solfataricus* was cultivated at 80°C by repeated additions of phenol at concentrations up to 400 mg.L^−1^, this level being shown to be under the inhibition threshold [18]. Flask cultures (500 mL) of phenol-adapted *S. solfataricus* 98/2 were used to inoculate the fermentor filled with standard mineral medium with phenol (initial optical density (OD) in a range of 0.15–0.20). Oxygen was first fed to maintain a dissolved-O_2_ concentration (DOC) of 1.5 mg.L^−1^. When biomass reached about 0.35 g.L^−1^, the oxygen set up was decreased to a DOC of 0.06 mg.L^−1^. In all experiments, stirring was adjusted to 300 rpm and total aeration-flow rate to 100 mL.min^−1^. Under these conditions, the K_L_a value for oxygen was 82.8 h^−1^ [17]. DOC was regulated through the O_2_/N_2_ ratio in the inlet gas. The pH was maintained at 3.2 with NaOH 0.5 mM. The exit gas was efficiently cooled to avoid phenol loss by evaporation as described elsewhere [17]. For proteomic and transcriptomic studies, experiments were also carried out on glucose (1.8 g.L ^−1^).

### Analytical methods

Cell density was determined by OD measurement at 600 nm. Cell dry weight was calculated from OD data by using the relation of 1 OD unit  =  320 mg.L^−1^ [17].

Phenol consumption and intermediate-metabolite production were followed, after centrifugation of the sample (5 min, 14000 rpm), by HPLC equipped with a Diode Array Detector (DAD), as previously described [17]. Intermediary-metabolite concentrations are expressed as mg_eq.phenol_.L^−1^. Chemical structures of intermediates were explored by Liquid Chromatography Mass Spectrometry (LC-MS) using a Hitachi Elite LaChrom L-2130 liquid chromatograph coupled to a Bruker Esquire 6000 MS. The MS detector is equipped with an electro spray ionization in positive and negative mode and a quadrupole analyzer. Separation was achieved with a Varian Polaris C18 column eluted by a gradient of acetonitrile in water containing acetic acid (0.1% v/v), from 0 to 40% acetonitrile during 20 min, at a flow rate of 200 µL.min^−1^. The cone and capillary voltages were maintained at 30 and 3500 V, respectively. To determine those chemical structures, 10 mL of culture were centrifuged; the supernatant extracted with ethyl acetate and analyzed by GC-MS as already described [33].

Both biomass and phenolic-compound analyses were made by triplicate and the average value was reported. Specific growth rate (µ, h^−1^) and specific degradation rate (q_P_, mg.g^−1^.h^−1^) were calculated from biomass and phenol concentration data.

Oxygen consumption was calculated through the O_2_ mass flow needed to maintain the DOC set point in the broth. Carbon dioxide production was measured online in the exit gas by infrared analyzer. The oxygen yield factor (Y_X/O2_, g.g^−1^), the respiratory quotient (Q_resp_, mol.mol^−1^), the biomass yield on phenol (Y_X/P_, g.g^−1^) and the carbon balance were calculated as previously described [17].

### RNA extraction

Cells of *S. solfataricus* (50 mL), grown in different conditions, were harvested by centrifugation 10 min at 7400 rpm at 4°C. Pellets were snap-frozen in liquid N_2_. RNA was extracted from the cell pellets using the High Pure RNA Isolation Kit (Roche Applied Science, USA). The purified nucleic acids were then treated with Turbo DNase (Ambion, USA) and RNA were purified again with the same kit. The quantity and quality of the obtained RNA were evaluated spectrophotometrically on a BioSpec-nano spectrophotometer (Shimadzu, Kyoto, Japan) and samples were diluted to 20 ng. µL^−1^ before conservation at -80°C.

### Semi-quantitative reverse-transcription PCR (RT-PCR) analyses

For RT-PCR analysis, total RNA (0.2 μg) was used to synthesize cDNA by triplicate in 20-µL reactions using the SuperScript® III Reverse Transcriptase and random primers (InVitrogen, USA). PCR was performed for 15, 20, 25 or 30 cycles using a step-cycle program of 98°C for 30 s, 50°C for 30 s, and 72°C for 20 s. Primers were designed using the Primer3 software. Primer pairs are listed Table 1. The amplification products were separated on a 1% agarose gel by electrophoresis, and the gel images were acquired using a Gel Doc Imager (Biorad, USA). DNA controls were carried out to exclude any DNA contamination.

**Table 1:** Primers used in gene-expression analyses in *S. solfataricus* 98/2.

Genes	Proteins	Primers
*ssol*_0230	4-hydroxyphenyl pyruvate dioxygenase	5′-CACTGTGGCCAAGTTTCTGA-3′
		5′-CCATAACGCTTTTGGGATGT-3′
*ssol*_1707	Gentisate 1,2 dioxygenase	5′-AGGGGACTAACGCCTACGAT-3′
		5′-AAACATCACCTGCCTTCCAC-3′
*ssol*_2369	Homogentisate 1,2 dioxygenase	5′-TACGCATCCTTTTGACGTTG-3′
		5′-GGAACAGACTGGGGGTGATA-3′
*ssol*_2712	Extradiol cleavage dioxygenase	5′-CGGTTTCCCAGAAGAGACCT-3′
		5′-GGGCTATTGTCGGTAATGGA-3′
*ssol*_2912	Catechol 2,3 dioxygenase	5′-TGCGCCTAATTTCTGTCTGA-3′
		5′-ATTGGGAGCCAATAGTGTGG-3′

### 2D-DIGE

The cells were harvested by centrifugation at 7000 g at 4°C for 15 minutes and washed with ice-cold phosphate buffered saline. The cells were broken by sonication in urea lysis buffer (8 M urea, 2 M thiourea, 4% (w/v) 3-[(3-Cholamidopropyl) dimethyl ammonio]-1-propane sulfonate (CHAPS), pH 8.5). The samples were then clarified by ultracentrifugation at 35000 g at 4°C for 1 h. Soluble proteins were purified and concentrated by precipitation with 4 volumes of ice-cold acetone, and solubilized for 1 h in 100 µL urea lysis buffer. The protein concentration was estimated using Bradford assay (Biorad, Hercules, CA, USA) according to the manufacturer’s instructions. All samples were then washed with the 2D-Clean-up kit (GE Healthcare, USA) and solubilized in urea lysis buffer to a final concentration of 2.5 µg. µL^−1^. The soluble *S. solfataricus*-protein fractions were labeled with cyanine dyes: Cy3, Cy5, Cy2 (CyDyes, GE Healthcare, USA). 25 µg of each protein extract was labeled separately at 4°C in the dark for 30 min with 200 pmol. µL^−1^ of the N-hydroxysuccinimide esters of cyanine dyes (Cy3 or Cy5). The internal standard, corresponding to a pool of the samples (12.5 µg of each individual extract), was prepared in parallel and labeled with Cy2. Total protein labeled with Cy2, Cy3 and Cy5 were combined and mixed with an equal volume of 2x urea lysis buffer containing 1% carrier ampholytes pH 4–7 according to the manufacturer’s instructions. For the first dimension (IsoElectric Focusing, IEF), precasted IPG (Immobilized pH Gradient) strips were used (pH 4–7, non linear (NL), 11 cm length; Immobiline DryStrips, GE Healthcare, USA). Typically, 75 µg of protein (25 µg for each dye) was loaded onto each IPG strip and the IEF was carried out (IPGPhor III, GE Healthcare, USA). The IEF protocol was as follows: 0–300 V gradient for 1h; 300–1000V gradient for 1.5 h; 1000–6000 V gradient for 2 h; 6000 V for 2 h. Temperature was set up at 20°C. Prior to SDS PAGE, IPG strips were equilibrated during 20 min in an equilibration buffer (6 M urea, 50 mM Tris pH 8.8, 2% SDS, 38.5% glycerol) added with 65 mM DTT for the first 10 min and 2% iodoacetamide for the further 10 min. The second dimension was performed using a Criterion Dodeca Cell separation unit (Biorad, Hercules, CA, USA) and precast 10% SDS-PAGE gels (Biorad, Hercules, CA, USA). IPG strips were placed on the top of the precast gels, overlaid with 0.5% agarose in 2x running buffer containing bromophenol blue. Gels were run at 20**°**C using the following XT-MES running buffer (Biorad, Hercules, CA, USA): 1X for the cathode and 2X running buffer for the anode part. Electrophoresis was conducted overnight at 15 V and stopped when the bromophenol-blue-dye front has reached the bottom of the gel. After SDS PAGE, cyanine-dye-labeled-protein gels were scanned directly using the Typhoon FLA9000 scanner (GE Healthcare, USA). All gels were scanned with a resolution of 50 µm. Determination of protein abundance and statistics based on 2D DIGE were carried out with the Decyder Software (version 6.5, GE Healthcare, USA). First step for the spot detection is the creation of crop images of the region of interest. Cropped images were imported onto Decyder. Spot detection was set as 10000 with a filter volume set at 30000. Spot selection was performed for a ratio up to 2 and a t-test p-value < 1%.

### Gel digestion and MALDI-TOF MS

MALDI-TOF MS is based on the Decyder analysis. Spots of interest were excised using Shimadzu Biotech Xcise System (Champs sur Marne, France). The proteins were subjected to in-gel digestion with trypsin, (Sequencing-grade modified porcine trypsin; Promega, Madison, WI, USA). Tryptic peptides were then extracted from the gel by successive treatment with 5% formic acid and 60% acetonitrile/5% formic acid. Each treatment is followed by sonication (5 min). Extracts were pooled and dried in a Speedvac evaporator. Peptides, resuspended in an α-cyano-4-hydroxycinnamic-acid-matrix solution (prepared by diluting 6 times a saturated solution in 50% acetonitrile/0.3% trifluoroacetic acid), were then spotted on the metal target. Mass analyses were performed on a MALDI-TOF Bruker Ultraflex III spectrometer (Bruker Daltonics, Wissembourg, France) controlled by the Flexcontrol 2.0 package (Build 51). This instrument was used at a maximum accelerating potential of 25 kV and was operated in reflector mode and the m/z ranges from 600 to 3500. Six external standards (Peptide Calibration Standard II, Bruker Daltonics, Wissembourg, France) were used to calibrate each spectrum to a mass accuracy within 10 ppm with a minimal resolution of 10000 the angiotensin II peak (Monoisotopic mass  =  1046.542 Da). Peak picking was performed with Flexanalysis 2.0 software (Bruker Daltonics, Wissembourg, France) with an adapted-analysis method. Parameters used were as follows: SNAP peak detection algorithm, S/N threshold fixed to 6 and a quality factor threshold of 30. A list of contaminations was constituted from a blank sample (blank piece of gel treated and analyzed exactly as a true sample). The trypsin peaks of this blank sample were excluded of the database search.

### Protein identification

Database searches for MS spectra were conducted using MASCOT software 2.2 (Matrix Science), available online, against the *S. solfataricus* P2-complete-proteome NCBI database for known proteins. Peptide-mass tolerance was set to 100 ppm. Criteria used for protein identification are given by Mascot as a Probability Based Mowse Score. Ion score is -10*Log (P), where P is the probability that the observed match is a random event. Protein scores greater than X (X is a number between 55 and 74, from one search to the other) are significant (p<0.05). The matching of theoretical pI and PM of the identified protein with those observed in the 2D-gel experiments was also used as another criterion of confidence.

## Results

### Growth kinetics and yields, degradation products and carbon balance

**Figure 1:**
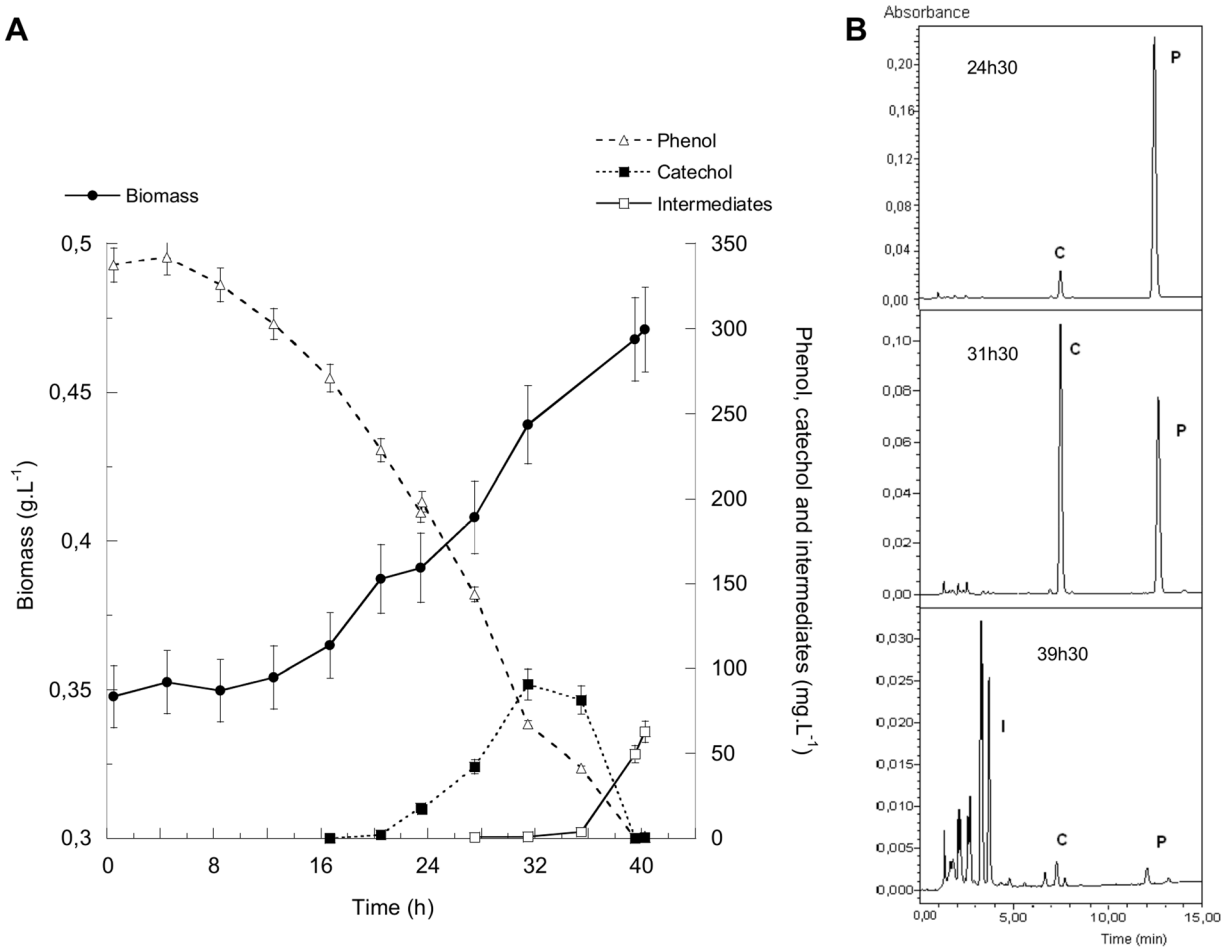
Analysis of *S. solfataricus* 98/2 cultures grown on phenol at a DOC of 0.06 mg.L^−1^. (A) biomass, catechol and other intermediate productions and phenol consumption. The intermediate curve corresponds to the sum of all the detected compounds. (B) HPLC chromatograms (λ =  270 nm) obtained at three different time course of the run (P: phenol, C: catechol, I: intermediates).

As suggested previously, modifying the operating conditions for culture, such as DOC, can be a way to identify the phenol degradation pathway through the apparition of intermediary metabolites [27]. When *S. solfataricus* was grown with a DOC of 1.5 mg.L^−1^ after two sequential phenol batches (phenol initial concentration < 400 mg.L^−1^), biomass reached a concentration of 0.35 g.L^−1^ within 4 days (data not shown). At the third phenol addition (350 mg.L^−1^), the DOC was drastically decreased to 0.06 mg.L^−1^. Kinetic and respirometric yields and rates are presented in Table 2. Although cells were repeatedly grown on phenol (no lag phase was observed at 1.5 mg.L^−1^), at the lower O_2_ level, a short adaptation period was needed for growth and phenol consumption (Figure 1A). Then, biological activity started again and was characterized by aµ of 0.0106 h^−1^ and a q_p_ of 29.0 mg.g^−1^.h^−1^. These parameters were lower than those observed at 1.5 mg.L^−1^ (µ  =  0.0235 h^−1^ and q_p_  =  47.5 mg.g^−1^.h^−1^, respectively (Table 2)). In the same way, when the O_2_ concentration decreased, so did the Y_X/P_ (from 0.539 to 0.426 g.g^−1^).

**Table 2:** Kinetic and respirometric rates and yields of phenol degradation by phenol-grown *S. solfataricus* 98/2 cells at two dissolved-oxygen concentrations (DOC).

Parameters		DOC	
		1.5 mg.L^−1^	0.06 mg.L^−1^
µ (h^−1^)		0.0235 (R^2^ = 0.996)	0.0106 (R^2^ = 0.995)
q_p_ (mg.g^−1^.h^−1^)		47.5 (±1.4)	29.0 (±1.1)
Y_X/P_ (g.g^−1^)		0.539 (±0.023)	0.426 (±0.057)
Q_resp_ (mol.mol^−1^)		0.828 (±0.008)	0.805 (±0.005)
Y_X/O2_ (g.g^−1^)		0.415 (±0.005)	0.265 (±0.035)
Carbon balance (%)		97.4 (±5.5)	103.1 (±0.7)
	- biomass* (%)	34.3 (±1.5)	27.2 (±3.7)
	- carbon dioxide (%)	63.1 (±4.0)	60.2 (±2.9)
	- metabolites (%)	0	15.7 (±0.2)

_1.8_O_0.5_N_0.2_ and a molecular weight of 24.6 g.mol^−1^ [15]. on the basis of a phenol-grown biomass empirical formulae of CH

At DOC of 0.06 mg.L^−1^, carbon dioxide production and redox potential profiles were similar to those observed at 1.5 mg.L^−1^, i.e. O_2_ consumption and CO_2_ production increased and redox potential decreased with growth (data not shown). This has already been described [17]. However, the yield coefficient for oxygen (Y_X/O2_) was 0.415 g.g^−1^ for a DOC of 1.5 mg.L^−1^ and decreased significantly to 0.265 g.g^−1^ for a DOC of 0.06 mg.L^−1^. The respiratory quotient (Q_resp_) was closed to the theoretical value of 0.87 mol.mol^−1^ and was not affected by the oxygen level (Table 2).

At 0.06 mg.L^−1^, during phenol consumption, catechol – identified with a standard solution by its absorption spectrum (λ_max_  =  275 nm) and its retention time (7.20 min) in HPLC/DAD analysis – appeared in the broth after 20 h (Figure 1). Catechol reached a maximum concentration of 90 mg_eq.phenol_.L^−1^ at 32 h and then decreased (Figure 1A). Its dissimilation is correlated with the appearance of various, but minor, compounds, also evidenced by HPLC/DAD. Only three of the peaks corresponded to compounds with a defined λ_max_. They are characterized by retention times of 2.55, 3.2 and 3.6 min, respectively, with the analysis conditions given in the Material and Methods section. They displayed a λ_max_ of 290, 254 and 265 nm, respectively (Figure 1B). Trace amounts of them appeared after 28 h (Figure 1A) and their concentration increased strongly when catechol concentration decreased (36 h). At the end of the run (40 h), they reached a maximum concentration of 6.4, 47.4 and 12.3 mg_eq. phenol_.L^−1^, respectively.

LC/MS analysis formally confirmed the presence of catechol in all the samples withdrawn after 20 h of run, but not the *cis,cis* muconic acid (λ_max_  =  260). In the same way, 4-OC, belonging to the *meta* pathway, was also identified.

Moreover, at the same time that the intermediate metabolites are detected (28 h), a yellow color appeared instantaneously in the samples in contact with air. Its intensity grew with time. In contrast, none of the samples at 1.5 mg.L^−1^ displayed this phenomenon.

At both DOC, carbon balances are closed to 100% (Table 2). However, at 1.5 mg.L^−1^, carbon from phenol is exclusively directed toward CO_2_ (63.1%) and biomass (34.3%), while at 0.06 mg.L^−1^, carbon distribution is different. In this case, the CO_2_ proportion is maintained relatively constant (60.2%) while the biomass part decreased (27.2%) and a significant intermediary metabolite amount was observed (15.7%).

### Genomic analyses

Few studies concerned the enzymes involved in phenol degradation through the *meta* or *ortho* pathways in Sulfolobales. Chae et al. [31] have clearly identified the presence of a C23D-coding gene in *S. solfataricus* 98/2 through PCR amplification, sequencing and heterologous production in *E. coli* (Accession number EF494887). This enzyme showed the highest activity against catechol and 4-chlorocatechol. The corresponding *orf* was not identified in the genome annotation (Accession number NC_017274.1). Using a primer pair designed on the *S. solfataricus* P2 C23D genomic sequence (*sso*1223), we were able to amplify a fragment which sequence perfectly matched the one amplified by Chae et al. [31]. We decided to name this *orf*, according to the *S. solfataricus* 98/2-genome annotation, *ssol*_2912 (KF701465). No other gene coding for protein potentially involved in the *meta* pathway were identified in the genome of *S. solfataricus* 98/2. However, 4 genes coding for putative C12D, involved in the *ortho* degradation pathway, were found in the genome: *ssol*_0230 (4-hydroxyphenyl pyruvate dioxygenase), *ssol*_1707 (gentisate 1,2 dioxygenase), *ssol*_2369 (homogentisate 1,2 dioxygenase) and *ssol*_2712 (extra diol ring clivage dioxygenase).

### Transcriptional analysis

**Figure 2:**
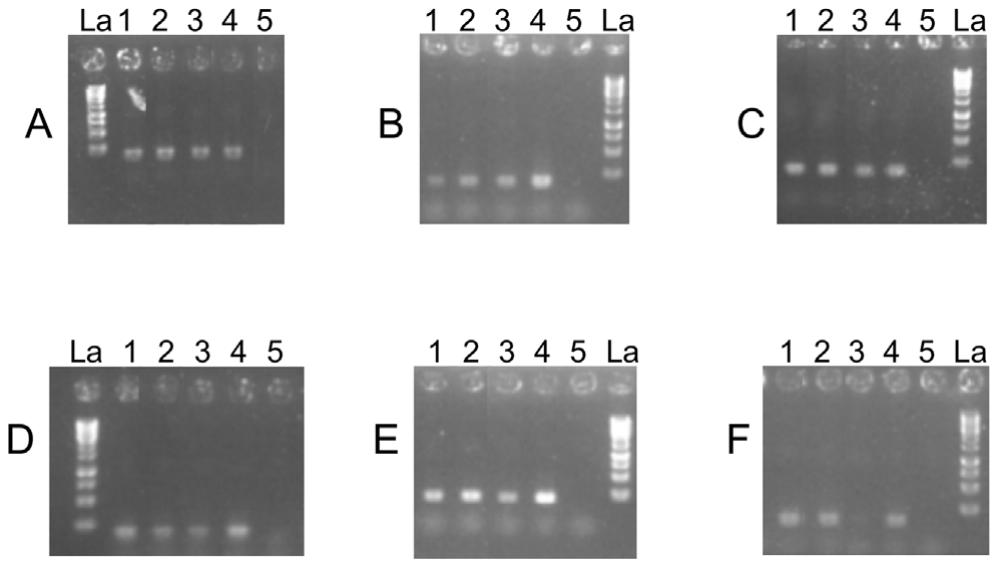
RT-PCR analyses. (A) 16S rRNA, (B) *ssol*_0230 (4-hydroxyphenyl pyruvate dioxygenase), (C) *ssol*_1707 (gentisate 1,2 dioxygenase), (D) *ssol*_2369 (homogentisate 1,2 dioxygenase), (E) *ssol*_2712 (extra diol ring cleavage dioxygenase), and (F) *ssol*_2912 (C23D) genes from *S. solfataricus*. Amplified fragments are 200-bp long. Lane 1: cells of *S. solfataricus* cultivated with phenol and a DOC of 0.06 mg.L^−1^. Lane 2: cells of *S. solfataricus* cultivated with phenol and a DOC of 1.5 mg.L^−1^. Lane 3: cells of *S. solfataricus* cultivated in glucose and a DOC of 1.5 mg.L^−1^. Lane 4: positive control, PCR amplification on genomic DNA as template. Lane 5: negative control experiment performed omitting reverse transcriptase during RT-PCR reaction and showing complete absence of DNA in the RNA samples. La: 1 kb Ladder (Fermentas, France).

To investigate which nutrient conditions induce the transcription of these different oxygenases in *S. solfataricus*, the transcription levels of these genes were examined by semi-quantitative RT-PCR analysis. For this analysis, mRNA were isolated from samples of *S. solfataricus* grown in liquid media with glucose or phenol as the sole carbon source and different oxygen concentrations. RT-PCR controls, using primer pairs designed to amplify 16S rRNA gene, showed the same expression level in each culture condition (Figure 2A). Using the primer pair designed to amplify *ssol*_0230, *ssol*_1707, *ssol*_2369, *ssol*_2712 and *ssol*_2912 genes, a unique band of the expected size was obtained (Figure 2B-F). The mRNA transcription levels of *ssol*_0230 were equivalent when the strain was harvested from the medium with glucose or phenol and with a DOC of 1.5 mg.L^−1^ (Figure 2B, lanes 2-3), but decreased when the strain was cultivated with phenol at a DOC of 0.06 mg.L^−1^ (Figure 2B, lane 1). The mRNA transcription levels of *ssol*_1707 were equivalent with low and high oxygen concentrations in presence of phenol (Figure 2C, lanes 1-2) and slightly lower when the strain was harvested from the medium with glucose as substrate (Figure 2C, lane 3). The mRNA transcription levels of *ssol*_2369 were equivalent in the strain cultivated with glucose and phenol (Figure 2D, lanes 2-3). However, it increased when the strain was harvested from the medium with phenol at a DOC of 0.06 mg.L^−1^ (Figure 2D, lane 1). The mRNA transcription level of *ssol*_2712 was slightly lower when the strain was harvested from the medium with glucose compared to phenol (Figure 2E, lanes 2-3). Yet, the transcriptional level of the extra-diol-ring-clivage-dioxygenase gene was higher in the media with high oxygen concentration (Figure 2E, lanes 1-2). The mRNA transcription level of *ssol*_2912 was undetectable when the strain was harvested from the medium with glucose as the sole carbon source (Figure 2F, lane 3). Nevertheless, it greatly increased when the strain was harvested from the media with phenol (Figure 2F, lanes 1-2). This differential expression of the C23D gene was detected in the medium whatever the DOC.

### Proteomic analysis

**Figure 3:**
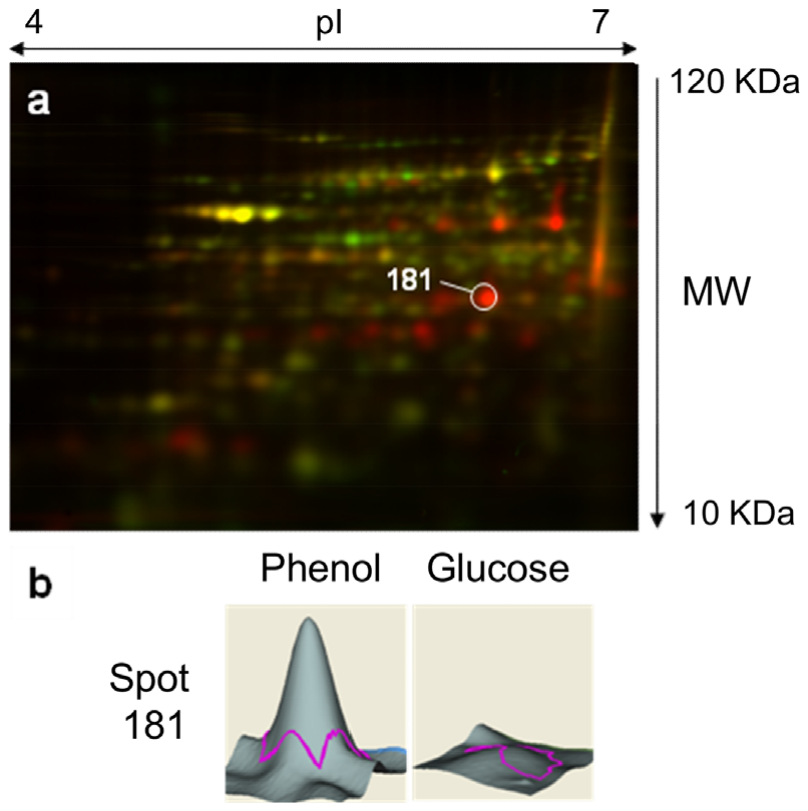
Differential proteome of *S. solfataricus* 98/2 grown on phenol or glucose. (A) 2D-DIGE gel. Cy2 (yellow): glucose + phenol conditions, Cy3 (green): glucose condition, and Cy5 (red): phenol condition. (B) Relative intensity of spot 181.

To investigate the influence of phenol on protein production in *S. solfataricus* 98/2, a 2D-DIGE experiment was performed. We compared the whole protein content of the cells grown on phenol or glucose. The representative 2D-DIGE gel is shown in Figure 3A. Interestingly, among the proteins more abundant on phenol only, spot 181 (Figure 3A) was identified as the C23D by MALDI-TOF MS. This enzyme is only detected on phenol (Figure 3B). Besides, no other dioxygenase could be identified as produced more abundantly on phenol. In particular, no differential production was observed for the putative C12D (Ssol_0230, Ssol_1707, Ssol_2369 and Ssol_2712).

## Discussion

Phenol and its derivatives are widely distributed environmental pollutants that are responsible for many unhealthy effects on humans. Degradation of such compounds has become of increasing interest for many years. As pointed out by Cao et al. [10], the most effective and economical way to remove them from the environment is the microbiological way. The catabolism of phenol involves the action of a monooxygenase responsible for the oxygenation of phenol to catechol. The latter is then subjected to either a *meta* or an *ortho* cleavage of the aromatic ring yielding 2-HMS or *cis*-*cis* muconic acid, respectively [2], [4], [8].

In this paper, we designed experiments to understand which pathway is involved in phenol degradation in *S. solfataricus* 98/2. One of the easiest ways to define the degradation pathway is to identify the reaction products. However, in *S. solfataricus* 98/2 cultivated in standard conditions, phenol is completely metabolized into CO_2_, H_2_O and biomass [17]. In their paper, Kapley et al. [27] observed that 2-HMS accumulated at low DOC (2 mg.L^−1^), probably because of a slower metabolism, which, in turn, allowed observing transitory accumulation of intermediary compounds. In our first experiment, performed at 1.5 mg.L^−1^, the growth parameters were determined. Nevertheless, in this condition and in contrary to what was expected, we were unable to detect any intermediate for the phenol degradation. Our experimental set up enables to work at a regulated and constant DOC. The problem of reducing the DOC to a very low level is that, is this case, the oxygen might become the limiting factor for the growth. Previous experiments showed that, with our experimental set up, for DOC as low as 0.06 mg.L^−1^, the carbon source still remains the limiting factor (data not shown). With such operating conditions, the behavior of the strain was notably modified. At this level, in comparison to the parameters measured at 1.5 mg.L^−1^, growth (µ) and phenol-biodegradation rates (q_p_) were slowed down (55% and 39%, respectively). These results can be compared to those reported by Ali et al. [28] who demonstrated that phenol specific degradation decreased with the oxygen concentration. Biomass yields on phenol (Y_X/P_) and on oxygen (Y_X/O2_) at 1.5 mg.L^−1^ are in the range of those reported in the literature (Table 2). For example, for two different *P. putida* strains cultivated on phenol in a continuous fed stirred-tank reactor, Seker et al. [34] and Nikakhtari and Hill [25] reported values of Y_X/P_ of 0.521 g.g^−1^ and 0.73 g.g^−1^, respectively and of Y_X/O2_ of 0.338 g.g^−1^ and 0.360 g.g^−1^, respectively. Feitkenhauer et al. [35] reported, with a *Bacillus thermooleovorans* strain, an Y_X/O2_ of 0.48 g.g^−1^. At 0.06 mg.L^−1^, we found that both parameters dropped 21% and 36%, respectively, indicating a less effective use of phenol or oxygen for biomass build up. Moreover, these coefficients (Y_X/S_ and Y_X/O2_) have already been shown to be sensitive to O_2_ levels for *S. solfataricus* as demonstrated by Simon et al. [36].

The slower growth of the strain (characterized by the 55% reduction of the growth rate), at a DOC of 0.06 mg.L^−1^, gave suitable conditions for the accumulation of some of the intermediates (15.7%), as confirmed by the carbon balance (Table 2). After 20 h of culture, catechol, resulting from the oxidation of the aromatic ring, appeared in the culture. It accumulated up to 90 mg.L^−1^ and decreased (Figure 1). Its consumption is correlated to the appearance of, at least, three other compounds. The λ_max_ of two of these compounds are characteristic of two intermediates of the *meta*-degradation pathway: the 2-hydroxymuconic acid (2-HMA, λ_max_  =  290 nm) from the 4-OC route and the 4-hydroxy-2-oxovalerate (HOV, λ_max_  =  265 nm) (Figure S1). Concomitantly, a yellow coloration is be detected in the sample after contact with air. As soon as the early seventies, Buswell and Twoney [37] reported the presence of this color during the degradation of phenol and cresol by a *Bacillus stearothermophilus* strain. This color has been reported as typical of the *meta* pathway biodegradation of monoaromatic compounds and associated with the presence of 2-HMS in thermophilic *Bacilli* strains [14], [38]. This has also been extensively reported in mesophilic strains (*Alcaligenes*, *Ralstonia*, *Pseudomonas*) [27], [39], [40]. The analysis of the last sample, using a more sensitive MS detector, strongly suggested the presence of 2-HMS. Moreover, the *cis*,*cis* muconic acid, characteristic of the *ortho* route, was not detected neither by UV_260nm_ nor by MS.

Dioxygenases are responsible for the opening of the ring. C12D open the ring between the C1 and C2 carbons of the catechol (*ortho* cleavage), while C23D open between C2 and C3 (*meta* cleavage). In the genome of *S. solfataricus* 98/2, four open reading frames putatively coding for 1,2 dioxygenases have been identified: *ssol*_0230 (4-hydroxyphenyl pyruvate dioxygenase gene), *ssol*_1707 (gentisate 1,2 dioxygenase gene), *ssol*_2369 (homogentisate 1,2 dioxygenase gene), and *ssol*_2712 (extra diol ring clivage dioxygenase gene). The expression of these genes has been semi-quantitatively followed depending on the growth conditions. Even if the expression of some of these genes seems to be oxygen dependant (*ssol*_0230 and *ssol*_2369), it is worth noting that all of the genes were expressed whether the carbon source was glucose or phenol. One open reading frame putatively coding for a C23D was identified in *S. solfataricus* 98/2 genome: *ssol*_2912 (C23D gene). The semi-quantitative analysis of its expression showed that, in presence of glucose, the transcript was not detectable indicating a regulation of the expression depending on the carbon source. No difference observed in the expression of the gene coding for 16S rRNA validated the approach.

To confirm the presence or the absence of the protein depending on the growth condition, a comparative analysis of the proteome between cells harvested on glucose or on phenol was performed. The only detectable difference among the dioxygenases is on the production of the C23D. The four C12D were not identified in this experiment. However, their theoretical pI being within the tested range (Ssol_1707: 6.11, Ssol_2369: 6.24, Ssol_0230: 6.37 and Ssol_2712: 5.06, respectively), their absence means that the four proteins are equally produced on glucose and phenol.

In conclusion, our set of results (transcriptomic and proteomic) seems to indicate that both degradation pathways are functional in presence of phenol. However, the activation of the C23D, only when phenol is present, and the accumulation of only intermediary compounds related to this pathway lead us to the conclusion that the aromatic ring is preferentially opened through the *meta* pathway.

## Supporting Information

Figure S1 **Phenol degradative pathways.** Dot arrow, *ortho* pathway. Dash arrow, *meta* pathway. MO, monooxygenase; C12D, catechol 1,2 dioxygenase; C23D, catechol 2,3 dioxygenase; 2-HMS H, 2-HMS hydrolase, 2-HMS DH, 2-HMS dehydrogenase; 4OT, 4-OC tautomerase; 4OD, 4-OC decarboxylase; OE H, OE hydratase; 2-HMS, 2-hydroxymuconic semialdehyde; 2-HMA, 2-hydroxymuconic acid; 4-OC, 4-oxalocrotonate; OE, 2-oxopent-4-dienoate; HOV, 4-hydroxy-2-oxovalerate; TCA: Tricarboxylic acid (adapted from Omokoko et al. [11]).
(TIF)
