# Supplementary figures and images for: Biochemical, Transcriptional and Translational Evidences of the Phenol-meta-Degradation Pathway by the Hyperthermophilic Sulfolobus solfataricus 98/2
Source: PLoS One. 2013 Dec 11;8(12):e82397. doi: 10.1371/journal.pone.0082397 (PMC3859572; doi:10.1371/journal.pone.0082397)

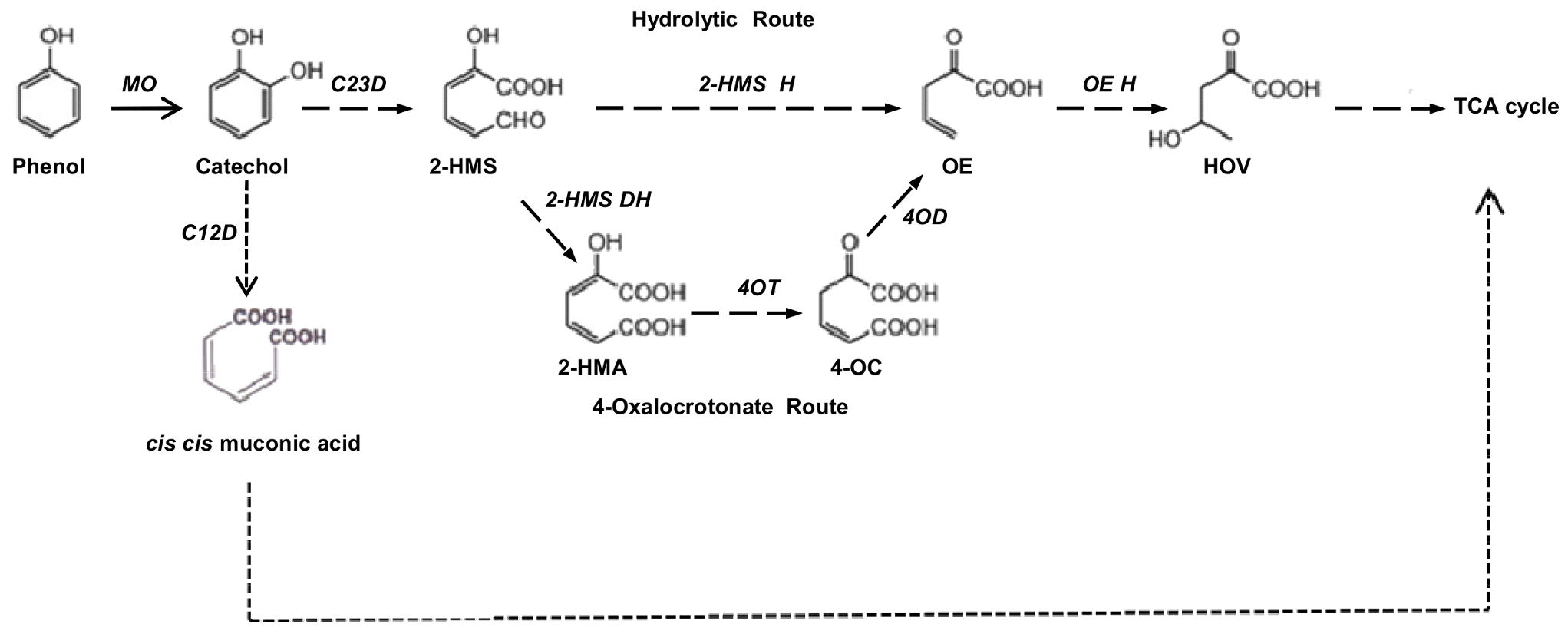

Supplement: Figure S1 — Phenol degradative pathways. Dot arrow, ortho pathway. Dash arrow, meta pathway. MO, monooxygenase; C12D, catechol 1,2 dioxygenase; C23D, catechol 2,3 dioxygenase; 2-HMS H, 2-HMS hydrolase, 2-HMS DH, 2-HMS dehydrogenase; 4OT, 4-OC tautomerase; 4OD, 4-OC decarboxylase; OE H, OE hydratase; 2-HMS, 2-hydroxymuconic semialdehyde; 2-HMA, 2-hydroxymuconic acid; 4-OC, 4-oxalocrotonate; OE, 2-oxopent-4-dienoate; HOV, 4-hydroxy-2-oxovalerate; TCA: Tricarboxylic acid (adapted from Omokoko et al. [11]). (TIF) [file pone.0082397.s001.tif]
